# Supplementary material for: Using protection motivation theory to support patient adherence in healthcare settings: A scoping review
Source: PLoS One. 2025 Sep 2;20(9):e0330883. doi: 10.1371/journal.pone.0330883 (PMC12404470; doi:10.1371/journal.pone.0330883)
Supplement: Attachment — Submitted filename: Response to Reviewers.docx [file pone.0330883.s002.docx]

Dear Editor,

Thank you for the opportunity to submit a revised draft of the manuscript “Using Protection Motivation Theory to support patient adherence in healthcare settings: A scoping review” for publication in PLOS ONE. We have incorporated most of the suggestions made by the reviewers and these are highlighted within the manuscript. Please see below a point-by-point response to the reviewers’ comments. All line numbers refer to the Revised Manuscript with Track Changes file.

Reviewer #1: Thank you very much for the opportunity to review this article. It provides many interesting insights, and I have a few small questions:

1. The introduction needs to better highlight the significance of the study; it seems insufficiently expressed in this regard.

*Thank you for highlighting this. We have amended the Introduction to better express the significance and aim of the study:*

***“Studies show adherence rates can be as low as 30% in unsupervised home exercise programs and vary significantly across conditions (****Ley and Putz 2024).” (Lines 45-46)*

***“Meta-analyses have extensively validated PMT across various health contexts, demonstrating moderate to strong relationships between PMT variables and health behaviours (d+ = 0.52), with particularly strong effects for self-efficacy (d+ = 0.88) and notably higher effect sizes (d+ = 0.98) for medical treatment adherence specifically (Floyd et al., 2000).”*** *(Lines 53-56)*

***“While PMT's theoretical validity is well-established, uncertainty remains around how to effectively translate it into clinical practice.”*** *(Lines 71-72)*

***“Our objective is not to re-validate PMT but rather to systematically map how PMT is being applied to improve adherence in physical healthcare contexts, providing practical guidance for frontline health professionals seeking to implement PMT-based interventions in routine care.”*** *(Lines 77-81)*

2. There are several technical terms in the article that appear to be self-explanatory, such as "threat appraisal" and "coping appraisal." I believe the authors should provide more explanation for these terms.

*We agree with the reviewer’s comment and have added some further explanation of these terms in the Introduction (Lines 58-62):*

*“Threat appraisal refers to an individual’s perception of their vulnerability to, and severity of, the health threat, as well as any intrinsic or extrinsic rewards associated with maladaptive behaviours. Coping appraisal refers to an individual’s perception of their ability to carry out the recommended protective behaviour change (self-efficacy), the effectiveness of the behaviour to reduce the threat (response efficacy), and the response cost required to perform the behaviour.”*

3. The article mainly focuses on a simple description of previous studies. Although I was looking forward to seeing a classification of the PMT theoretical framework or some other kind of organization, it seems this is missing.

*In the scoping review we have focused on interventions that have drawn on PMT to increase patient adherence in healthcare settings. PMT therefore provides the theoretical framework for the review. We were unclear whether the reviewer also wanted us also to classify the PMT (and in what way). PMT is a model of the social cognitive determinants of health behaviour (Conner & Norman, 2015) that, for example, predominantly focuses on the motivation component of the COM-B model of behaviour change (Michie et al., 2011). We have not included this information in the revised submission, but would be happy to do so if the reviewer feels that it is important to do so.*

4. Additionally, some previous research has highlighted the impact of cultural context on PMT. Given that the sample size in this study is not large, I think the article could benefit from further exploration and extension from a cultural perspective.

*We agree that the impact of cultural context on PMT is an important consideration. We feel that an in-depth exploration of this goes beyond the scope of this review, however, we have added some additional details on this in the manuscript.*

*Firstly, we have added the country of origin to the Results (Lines 141-2) and in Table 1:*

*“Studies included in the review were conducted in a range of countries: USA (n=4); Iran (n=3); China (n=3); Canada (n=1), New Zealand (n=1), and the UK (n=1).”*

*Secondly, the Discussion identifies a lack of generalisability to lower income countries as a limitation of the review (Lines 251-2).*

*“Generalisability is limited given that all interventions were trialled in upper-middle- or high-income countries”.*

*Thirdly, we have commented on the consideration of cultural context in the Discussion (Lines 260-1):*

*“Cultural factors should be taken into account when designing and adapting interventions to ensure that these are relevant for the target audience.”.*

Reviewer #2:

1. The significance of the research is not prominent enough. Although the research topic is interesting, it needs to be emphasized why this topic is important and worth studying.

*Thank you for this comment. The need to emphasise the significance of the research was also raised by Reviewer 1. We believe we have addressed this comment in our response to Reviewer 1’s first comment.*

2. While the eligibility criteria are detailed and the sample inclusion appears well-defined, I wonder if 13 studies are sufficient to support a comprehensive review. For researchers familiar with Protection Motivation Theory (PMT) in the context of patients and health adherence, many would likely already be aware of these studies.

*We believe that the review makes a valuable contribution to knowledge in this area, even with only 13 studies. The review maps the existing evidence on the use of PMT-based interventions to improve patient adherence in healthcare settings and its results provide context-specific, actionable knowledge about the application of PMT.*

*The study is aimed at healthcare professionals who are interested in improving patient adherence in order to improve clinical outcomes. As such, the target audience would likely not be aware of these studies. In addition, synthesising the literature in this way adds value even for those who are aware of some of the individual studies as it provides an overview of the range of ways that PMT has been applied in this context. The target audience of the review has been clarified in the manuscript (Lines 79-80):*

*“…providing practical guidance for frontline health professionals seeking to implement PMT-based interventions in routine care.”*

3. Consider a Meta-Analysis on PMT: Would a meta-analysis on PMT offer greater value? For example, exploring whether conflicting relationships exist between perceived threat, perceived efficacy, and health behaviors could yield deeper insights.

*Existing meta-analyses have validated PMT across various health contexts. We sought not to re-validate PMT, but rather to map the ways that it has been applied to improve adherence in physical healthcare contexts. We opted to conduct a scoping review as these are designed to map the available literature on a topic, identify knowledge gaps, and summarise key findings across a variety of study types. They do not provide a quantitative synthesis of the data as a meta-analysis would. We hope that the amended introduction better explains this decision.*

*We agree that it would be valuable to understand whether any particular characteristics of the interventions were associated with those studies which found significant differences between intervention and control groups.* *The main difference between the studies that found significant vs non-significant differences appears to relate to whether they targeted the response efficacy construct. All of those studies which found significant differences targeted response efficacy, compared to 2/5 of those which did not find significant differences.*

*We have added the following text to the Results (Lines 213-5) to reflect this:*

*“Studies that found a significant difference in behaviour change between the intervention and control group more often targeted the response efficacy construct (7/7) than those which did not find significant differences (2/5).”*

4. Your appendix currently lists data collection and exclusion criteria in a basic format. It would be more informative to include the specific standards directly in this figure.

*We are unsure what the reviewer is referring to here as there is no appendix attached. If the reviewer would like to clarify, we would be very happy to respond.*

*We had meant to upload a supplementary file detailing the full search strategy with the original submission but it seems like this was missed. We have included this in the revised manuscript.*
