# Supplementary material for: Using protection motivation theory to support patient adherence in healthcare settings: A scoping review
Source: PLoS One. 2025 Sep 2;20(9):e0330883. doi: 10.1371/journal.pone.0330883 (PMC12404470; doi:10.1371/journal.pone.0330883)
Supplement: Attachment — Submitted filename: Response to Reviewers July 2025.docx [file pone.0330883.s003.docx]

Dear Editor,

Thank you for the opportunity to submit a revised draft of the manuscript “Using Protection Motivation Theory to support patient adherence in healthcare settings: A scoping review” for publication in PLOS ONE. We have incorporated the suggestions made by the reviewer and these are highlighted within the manuscript. Please see below a point-by-point response to the reviewers’ comments. All line numbers refer to the Revised Manuscript with Track Changes file.

1. Although the authors provide a description of the main constructs of Protection Motivation Theory (PMT), I recommend including a conceptual figure that visually represents these components. A clear diagram of the PMT model would help understand the theory’s core constructs (e.g., threat appraisal, coping appraisal) and how they are defined and operationalized across the reviewed studies.

We agree that this will be a helpful addition to the manuscript. We have now included Figure 1 which depicts the core constructs within PMT.

2. The manuscript would benefit from a clear explanation of why a scoping review was chosen over a systematic review or meta-analysis. A brief justification in the Methods or Introduction section would clarify the scope and intentions of the review for the reader.

Thank you for highlighting this. We have strengthened the justification for this choice in the final paragraph of the introduction:

*“Scoping reviews can be used to map concepts and research activity across a broad area to clarify methods and identify gaps to shape future studies. Our aim is not to assess effectiveness as in a systematic review, but to systematically characterise how PMT is being applied to improve adherence in physical healthcare contexts,” (Lines 76-78)*

3. One of the stated aims of the review is to provide ‘practical guidance for frontline healthcare professionals’ on applying PMT-based interventions. This aim should be more explicitly addressed in the Discussion section.

Thank you for this comment. We have made our reflections on this more explicit in the discussion:

“*The synthesis provides a breakdown of the existing PMT based interventions in a range of clinical areas, allowing healthcare professionals to select components that are relevant to and practicable in their setting”. (Lines 250-252)*

*“The most relevant constructs within PMT are likely to differ across health conditions and treatment regimens. Therefore, exploratory research should be undertaken prior to intervention development to identify the constructs most pertinent to the specific clinical context.” (Lines 255-257)*

Yours faithfully,

Liv Hawksworth
